# Association of *BRCA* Mutations and Anti-müllerian Hormone Level in Young Breast Cancer Patients

**DOI:** 10.3389/fendo.2019.00235

**Published:** 2019-04-11

**Authors:** Kyung-A Son, Dong-Yun Lee, DooSeok Choi

**Affiliations:** Department of Obstetrics and Gynecology, Samsung Medical Center, Sungkyunkwan University School of Medicine, Seoul, South Korea

**Keywords:** breast cancer, anti-Müllerian hormone, *BRCA1*, *BRCA2*, ovarian reserve

## Abstract

**Background:** Several preclinical and clinical studies have suggested that *BRCA*-mutation carriers may have decreased ovarian reserve. However, data in this area are limited and inconsistent, especially in young breast cancer patients.

**Objective:** This study evaluated the association between *BRCA* mutation status and serum anti-Müllerian hormone (AMH) level in young, reproductive-aged patients with breast cancer.

**Materials and Methods:** Patients ≤ 40 years of age with breast cancer and who had known *BRCA* status and baseline serum AMH level at Samsung Medical Center, Seoul, Korea, were considered for inclusion. A total of 52 *BRCA* mutation carriers (27 *BRCA1* and 25 *BRCA2*) and 264 non-carriers were selected for analyses. The serum level of AMH was compared according to presence of a *BRCA* mutation, and linear and logistic regression analyses were performed to evaluate the association between *BRCA* mutation and serum AMH level.

**Results:** No difference was found in clinical characteristics between *BRCA*-mutation carriers and non-carriers. Subjects with any *BRCA* mutation had a significantly lower median AMH than those without a mutation (2.60 vs. 3.85 ng/mL, 32% reduction, *P* = 0.004). Linear regression analysis showed a significant negative association between *BRCA* mutation and AMH level. In addition, logistic regression demonstrated non-significantly increased odds of mutation carriers having AMH < 1.2 ng/mL. However, no difference was found between *BRCA1/2* mutations.

**Conclusions:** Breast cancer patients with *BRCA* mutation have significantly lower serum AMH level. Fertility preservation should be considered more aggressively in young breast cancer patients with *BRCA* mutation.

## Introduction

*BRCA* mutations are associated with high risk of breast and ovarian cancer in reproductive-aged women (1, 2). The lifetime risks of breast and ovarian cancer are 65 and 39%, respectively, in *BRCA1* mutation carriers and 45 and 11% in *BRCA2* mutation carriers (3).

In addition to cancer risk, it has been suggested that *BRCA* mutation may be related to decreased ovarian reserve, due to BRCA's function in repairing double-strand DNA breaks (4). Several studies have demonstrated significantly decreased serum anti-Müllerian hormone (AMH) level, a biomarker representing ovarian reserves (5), in *BRCA* mutation carriers (6–9). Moreover, in breast cancer patients who underwent ovarian stimulation for fertility-preservation, there was a higher rate of poor ovarian response (POR) in *BRCA*-mutation carriers compared to non-carriers (10, 11). However, some studies have found no difference in serum AMH level according to *BRCA* mutation status (12–15). Therefore, the association between *BRCA* mutation status and decreased ovarian reserve is not conclusive. In addition, only a few studies have shown a significant association between *BRCA* mutation and decreased ovarian reserve in young breast cancer patients (16, 17).

Considering that it is currently recommended for *BRCA*-mutation carriers to complete childbearing by age 40 and to undergo a risk-reducing salpingo-oophorectomy, and that breast cancer patients with *BRCA* mutation are at increased risk of infertility as a result of anticancer treatment (18), issues of fertility preservation should be a priority for young patients.

Therefore, this study aimed to clarify the relationship between *BRCA* mutation and the level of ovarian reserve by comparing serum AMH level between *BRCA*-mutation carriers and non-carriers in breast cancer patients.

## Materials and Methods

This retrospective study included all premenopausal patients ≤40 years of age who were diagnosed with breast cancer and had a known baseline status regarding *BRCA* mutation and serum AMH level at Samsung Medical Center, Seoul, Korea, from December 2011 to May 2018. We excluded patients who (1) no longer had spontaneous menstruation at the time of tests, (2) had a history of any cancer treatment for breast cancer (i.e., chemotherapy or endocrine therapy), (3) had a history of another malignancy, (4) had a history of any ovarian surgery, (5) were pregnant, (6) had been diagnosed with any gynecologic problem that might affect AMH level (i.e., polycystic ovarian syndrome or endometriosis), and (7) had *BRCA* mutation of undetermined significance. Among the 316 patients included in this study, 264 were *BRCA*-negative and 52 were *BRCA*-positive (27 *BRCA1*-positive and 25 *BRCA2*-positive). The study was approved by the Institutional Review Board of Samsung Medical Center and exempted from informed consent requirements.

### Measurements

Serum AMH level was measured using AMH ELISA kits (Beckman Coulter, Fullerton, CA, USA) following the manufacturer's directions. The minimum detectable concentration was 0.16 ng/mL, and the inter- and intra-assay coefficients of variation were 5.6 and 5.4%, respectively.

*BRCA* testing was conducted on peripheral blood using direct sequencing. When pathogenic variants were identified in the genetic tests, all mutations were interpreted utilizing the Human Gene Mutation Database (HGMD; http://www.hgmd.cf.ac.uk/), ClinVar (https://www.ncbi.nlm.nih.gov/clinvar), and Korea ONCOgene Research and Diagnosis (KONCORD; http://koncord.kr). Mutation nomenclatures from the Breast Cancer Information Core (BIC; http://research.nhgri.nih.gov/bic/) were used for the genetic test reports.

### Statistical Analysis

Statistical analysis was executed using Statistical Analysis System software, version 9.4 (SAS Institute Inc., Cary, NC, USA).

Clinical characteristics and serum AMH level were compared based on the presence of *BRCA* mutation. Data are presented as median (interquartile range) or number (percentage). Differences between the groups were analyzed using Chi-square test or Fisher's exact tests for categorical variables and Student's *t*-test or Mann–Whitney *U*-test for continuous variables. A *P* < 0.05 was considered statistically significant.

Regression analyses were performed to reveal the relationships between serum AMH level and *BRCA* mutation status after adjusting for age, body mass index, and history of smoking and oral contraceptive use. Linear regression analysis was performed on log-transformed serum AMH levels due to the non-normal distribution of the AMH values. In addition, logistic regression analysis was conducted to examine the association between *BRCA* mutation status and low AMH level, which represents poor ovarian reserve. For analysis, AMH <1.2 ng/mL was considered as poor ovarian reserve based on a previous report (19).

## Results

Table 1 shows the clinical characteristics of the study subjects. The median age was 34 years for both the *BRCA*-positive and *BRCA*-negative groups. No differences were found in reproductive or menstrual history, and smoking and alcohol intake did not differ between the two groups. However, the proportions of patients who had progesterone receptor- or human epidermal growth factor receptor 2-positive cancer were significantly higher in the *BRCA*-positive than in the *BRCA*-negative group.

**Table 1:** Clinical characteristics of the study subjects.

	***BRCA*-negative (*n* = 264)**	***BRCA*-positive (*n* = 52)**	***P*-value**
Age, years	34.0 (30.0–36.0)	34.0 (30.5–36.0)	0.802
Body mass index, kg/m^2^	21.0 (19.5–23.0)	20.9 (19.3–24.0)	0.820
Age at menarche, years	14.0 (13.0–15.0)	14.0 (13.0–15.0)	0.863
Parity			0.635
0	148 (56.1%)	25 (48.1%)	
≥1	116 (43.9%)	27 (51.9%)	
Menstruation			
Regularity	199 (75.4%)	45 (86.5%)	0.064
Duration (days)	5.0 (5.0–6.5)	5.0 (4.0–6.5)	0.449
Infertility treatment history	7 (2.7%)	1 (1.9%)	0.760
Receptor status			
ER (+)	161 (61.0%)	27 (51.9%)	0.224
PR (+)	133 (50.4%)	18 (34.6%)	**0.038**
HER2 (+)	45 (17.1%)	0 (0.0%)	**0.001**
Smoking use	1 (0.38%)	0 (0.0%)	0.657
Alcohol use	2 (0.76%)	1 (1.9%)	0.428

*Data are presented as median (IQR) or number (%). Statistically significant differences are in bold. ER, estrogen receptor. PR, progesterone receptor; HER2, human epidermal growth factor receptor 2*.

**Figure 1:**
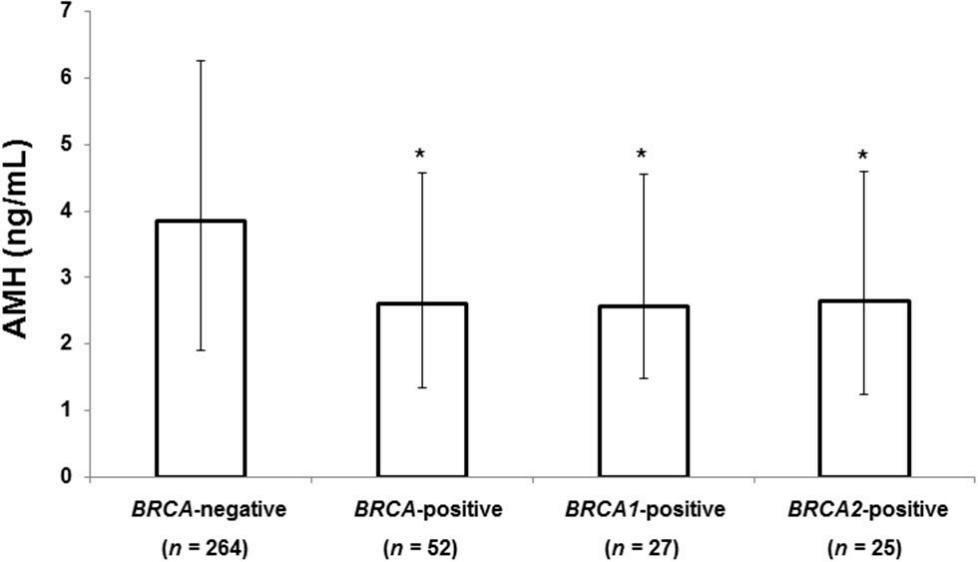
Median AMH level according to *BRCA* mutation status. The error bars indicate interquartile ranges. *P < 0.05 vs. *BRCA*-negative patients. AMH, anti-Müllerian hormone.

Figure 1 shows the median serum AMH level according to *BRCA*-mutation status. Patients with any *BRCA* mutation had a significantly lower median AMH than those without a mutation (2.60 vs. 3.85 ng/mL, 32% decrease, *P* = 0.004). Serum AMH levels of the *BRCA1* (2.56 ng/mL, *P* = 0.001) and *BRCA2* groups (2.64 ng/mL, *P* = 0.036) were significantly lower than that of *BRCA*-negative group, but no difference was found between the *BRCA1* and *BRCA2* groups.

Table 2 shows the results of linear regression analysis. Log-transformed AMH was negatively associated with age (*P* < 0.001). After adjusting for age, body mass index, and history of smoking and oral contraceptive use, serum AMH level was still significantly lower in the *BRCA*-positive group than in the *BRCA*-negative group (*P* = 0.043). Table 3 shows the results of the logistic regression model evaluating the association between risk of POR and *BRCA* mutation status. Thirty-five (13.3%) and 9 (17.3%) patients had AMH level <1.2 ng/mL in the *BRCA*-negative and *BRCA*-positive groups, respectively, presenting no statistical difference. After adjusting for age, body mass index, and history of smoking and oral contraceptive use, there was no increased likelihood of POR in the *BRCA*-positive group. In addition, no differences were found between the *BRCA1*- and *BRCA2*-positive groups in either the linear or logistic regression analysis (data now shown).

**Table 2:** Results of linear regression modeling of AMH level.

	**Parameter estimate**	**Standard error**	***P*-value**
Intercept	4.843	0.605	**<0.001**
BRCA 1/2 carrier	−0.309	0.152	0.043
BRCA non-carrier	(ref)		
**ADJUSTED VARIABLES**			
Age	−0.092	0.014	**<0.001**
Body mass index	−0.017	0.017	0.336
Smoking	0.517	1.004	0.607
Oral contraceptive use	0.067	0.359	0.853

*Regression of log transformed AMH level. Statistically significant differences are in bold. AMH, anti-Müllerian hormone*.

**Table 3:** The prevalence of poor ovarian reserve and the results of logistic regression model.

	***BRCA*-negative (*n* = 264)**	***BRCA*-positive (*n* = 52)**	***P*-value**
AMH level, number of patients (%)			0.441
<1.2 ng/mL	35 (13.3%)	9 (17.3%)	
≥1.2 ng/mL	229 (86.7%)	43 (82.7%)	
Odds ratio	(reference)	1.40	0.415
95% CI	(reference)	0.62–3.17	

*Adjusted for age, body mass index, and history of smoking and oral contraceptive use. AMH, anti-Müllerian hormone; CI, confidence interval*.

## Discussion

This study evaluated the association between *BRCA* mutation and serum AMH level in breast cancer patients aged ≤40 years. Median AMH was significantly lower in *BRCA*-positive breast cancer patients compared to *BRCA*-negative patients, but there was no difference in AMH level between the *BRCA1*-positive and *BRCA2*-positive groups.

Our results are similar to those of a previous study demonstrating a trend of lower AMH level (1.8 vs. 2.6 μg/L, *P* = 0.109) in 29 *BRCA*-positive breast cancer patients compared to 72 *BRCA*-negative breast cancer patients (17). Since age *per se* is an important factor determining serum AMH level, and patients with *BRCA* mutation show accelerated loss of ovarian follicular reserve and an earlier menopausal age (20), the differences in statistical significance in the studies might have resulted from inclusion of younger patients compared to our study (median age 31 vs. 34 years). Indeed, another study on patients with a median age of 34–36 years reported that AMH level was significantly lower in *BRCA*-positive breast cancer patients (1.22 vs. 2.23 ng/mL; *P* < 0.001) (16).

Several studies have shown that, in non-cancer, healthy subjects, serum AMH level was also significantly lower and ovarian follicles are fewer in *BRCA*-positive groups than in *BRCA*-negative groups (7–9). However, in one study, AMH levels were similar between 41 healthy *BRCA*-positive subjects and 324 healthy *BRCA*-negative subjects (12). Overall, the relationship between serum AMH level and *BRCA* mutation status remains contested.

When we analyzed the *BRCA1*-positive and *BRCA2*-positive groups separately in the present study, both had significantly lower AMH level than *BRCA*-negative patients, but no significant difference was found between the *BRCA*-positive groups. This finding is in accordance with previous studies presenting no significant difference in serum AMH level between *BRCA1*-positive and *BRCA2*-positive subjects (16, 17). In other studies, however, the serum AMH level was only significantly lower in either the *BRCA1*-positive (7) or *BRCA2*-positive (11) group compared to *BRCA*-negative subjects. Further studies are needed to evaluate the associations between each *BRCA* mutation and ovarian reserve.

In the current study, the prevalence of patients expected to exhibit POR, defined as AMH <1.2 ng/mL according to POSEIDON criteria (19), was not different according to *BRCA* mutation status, and the odds of AMH <1.2 ng/mL did not significantly increase after adjustment for age, body mass index, and history of smoking and oral contraceptive use. The results were the same when POR was assessed using an AMH level of <1.1 ng/mL (Bologna criteria) (21) or <1.0 ng/mL (17). Although the mean AMH of 2.64 ng/mL was lower in *BRCA*-mutation carriers than in non-carriers, the level should be sufficient for pregnancy due to the young age (34 years) of the patients in the current study.

The association between serum AMH level and *BRCA* mutation may be due to repair of double-strand DNA breaks and maintenance of chromosomal telomeres by BRCA (4, 22, 23). During reproduction, the telomere is shortened after every cycle of DNA replication, and telomere shortening is related to ovarian aging and reproductive lifespan (24). Furthermore, *BRCA1* gene expression decreases significantly with age in human oocytes. In a previous study, *BRCA1-*mutant mice had fewer oocytes after ovarian stimulation compared to wild-type mice and showed a tendency for DNA damage as a consequence of a deficiency in DNA double-strand break repair (16). Although BRCA2 also repairs DNA double-strand breaks, decreased *BRCA2* gene expression typically occurs at the end of the reproductive window, and the proportion of *BRCA2* gene expression among all DNA repair genes is small (25).

Our findings have substantial clinical importance in decision-making for young patients with breast cancer and *BRCA* mutation. From our results, *BRCA* mutation is an important factor associated with AMH level. Since fertility is attenuated with age, and a risk-reducing salpingo-oophorectomy should not be delayed over the long-term based on current recommendations, comprehensive, and individualized counseling for fertility preservation, such as oocyte or embryo cryopreservation, should be stressed in this population (26, 27).

This study has several strengths. First, the study population was relatively large (*n* = 316), and this is the largest reported study focusing on breast cancer patients. With the current sample size and difference in serum AMH level, a power of the current study is 98% with an alpha of 0.05. Second, our study evaluated the associations between *BRCA* mutations and decreased ovarian reserve in young breast cancer patients. Associations might differ between those who developed disease and those who were simply mutation-carriers, but most studies have only assessed healthy, non-cancer subjects. In addition, this is the first study of this kind in an Asian population. Genetic background differs across ethnicities; therefore, studies on various ethnicities are clinically important. For example, some studies (7, 12) have been performed on patients who carried at least 1 Ashkenazi Jewish founder mutation that is associated with a higher risk of breast and ovarian cancer (28, 29), and the results were different from ours. Finally, we analyzed the prevalence of POR according to *BRCA* mutation.

However, there are some limitations to our study. First, this was a retrospective study performed in one center. Second, we only analyzed serum AMH level to evaluate ovarian reserve. Although serum AMH level may be a reliable marker for ovarian reserve, addition of antral follicle count or serum follicle-stimulating level would be useful. Third, AMH is generally considered as the best ovarian reserve test, but it does not directly measure the primordial follicle pool. Fourth, although we addressed several factors that could affect serum AMH level, not all the potential confounders affecting serum AMH level were considered for analysis. Moreover, although we measured AMH and estimated POR, long-term fertility outcomes were not assessed in the present study.

In conclusion, young breast cancer patients with *BRCA* mutation have significantly lower AMH value, which is indicative of decreased ovarian reserve, compared to *BRCA*-negative patients. With further studies, our finding can support decision-making for fertility preservation.

## Ethics Statement

The study was approved by the Institutional Review Board of Samsung Medical Center and exempted from informed consent requirements.

## Author Contributions

K-AS, DC, and D-YL were responsible for the concept and design of the study, searching for and analyzing data, and the writing of the manuscript.

### Conflict of Interest Statement

The authors declare that the research was conducted in the absence of any commercial or financial relationships that could be construed as a potential conflict of interest.
